# First molecular detection of group A rotavirus in urban and hospital sewage systems by nested-RT PCR in Shiraz, Iran

**DOI:** 10.1186/2052-336X-11-4

**Published:** 2013-05-24

**Authors:** Mohammad Kargar, Negin Javdani, Akram Najafi, Yahya Tahamtan

**Affiliations:** 1 Department of Microbiology, Jahrom Branch, Islamic Azad University, Jahrom, Iran; 2 Department of Nursing and Midwifery, Firoozabad Branch, Islamic Azad University, Firoozabad, Iran; 3 Department of Biology, Payame Noor University, Tehran, Iran; 4 Department of Virology, The Persian Gulf Tropical Medicine Research Center. Bushehr University of Medical Sciences, Bushehr, Iran; 5 Razi Vaccine and Serum Research Institute, Shiraz, Iran

**Keywords:** Rotavirus, Sewage, Environmental surveillance, Genotyping

## Abstract

**Background:**

Group A rotaviruses are the most significant cause of acute gastroenteritis in children worldwide. Rotaviruses are shed in high numbers and dispersed widely throughout bodies of water in the environment. This represents a significant health hazard for humans, mainly due to the stability of the viruses during wastewater treatment processes. This study was conducted to evaluate the prevalence of rotaviruses, to determine G genotypes of circulating rotaviruses and to assess the efficiency of rotavirus removal in urban and hospital sewage treatment plants in Shiraz, Iran.

**Materials and methods:**

During the period from October 2010 to June 2011, a total of sixty sewage samples from urban and hospital sewage disposal systems were collected by Grab Sampling in Shiraz, Iran. All the samples were concentrated in pellet form and two-phase methods and then group A rotaviruses were investigated with enzyme immunoassays (EIA). Rotavirus-positive specimens were genotyped by the nested RT-PCR and by using different types of specific primers.

**Results:**

In total, rotaviruses were identified in 25% (15 cases) of sewage samples, representing 73.33% (11 cases) of influent and 26.67% (4 cases) of effluent systems. The frequency of rotavirus detection in autumn, winter and spring was 46.67%, 33.33% and 20%, respectively (*P*= 0.004). The most common circulating genotype was G1 (73.33%), followed by G1G4 (20%) and non-typeable (6.67%), respectively.

**Conclusions:**

The high prevalence of rotaviruses in urban and hospital sewage systems highlights the importance of environmental surveillance as a tool to detect new genotypes and to investigate the epidemiology of rotaviruses circulating in the community.

## Background

Despite major advances in current public health services and hygiene control in water and wastewater treatments, waterborne diseases still remain a potential risk to human health in both developed and developing countries [1]. Different types of enteric pathogens, including bacteria and viruses, have been implicated in outbreaks of waterborne gastroenteritis [1-4]. Rotaviruses are recognized as the most significant etiological agents of acute gastroenteritis in infants and young children worldwide [5]. Each year, these viruses are globally associated with 111 million cases of gastroenteritis, 25 million clinical visits, 2 million hospitalizations and 352,000-592,000 deaths among children aged <5 years old [6]. After replicating in the gastrointestinal tract, rotaviruses are shed in large quantities and may be disseminated widely into environmental waters such as groundwater [4], surface water [7], drinking water [8], and wastewater [3,9,10]. The stability of rotaviruses in different types of environmental water and their resistance to physicochemical treatment processes and adverse conditions in sewage treatment plants, makes their transmission through water highly significant [8,10,11]. It is important to consider the epidemiological surveys of rotavirus infection in water quality studies, not only because of their importance as the leading cause of severe diarrhea [5], but also because of their ability to survive in environmental waters for long periods of time [3,7] and the reuse of these waters for the irrigation of land used for crop cultivation [12]. Many studies have demonstrated the presence of group A rotaviruses in sewage and treated effluents as being responsible for 11 to 42% of all rotavirus-positive samples [7-9,11,13]. Despite the important role rotavirus infections play in childhood morbidity and mortality worldwide, very few hospital-based surveillance studies have been carried out in Iran. However, several epidemiological studies have shown that rotaviruses are a major cause in 27 to 46% of acute gastroenteritis cases in children <5 years old in Iran [14-16]. Until now, no data regarding the molecular epidemiology of group A rotaviruses in sewage and treated effluents systems in this region is available. The objectives of this study were to (i) evaluate the prevalence of rotaviruses (ii) determine the G genotypes of circulating rotaviruses (iii) assess the efficiency of rotavirus removal in urban and hospital sewage treatment plants in Shiraz, Iran.

## Methods

### Sampling

From October 2010 to June 2011, a total of thirty samples from urban sewage disposal systems and 30 samples from the Nemazee Hospital in Shiraz, Iran were collected by using the Grab Sampling procedure. The wastewater treatment plants of Shiraz cover an area the size of 72 hectares, which has a population of about 409,000 inhabitants. All the samples were obtained from the influent and effluent parts of the sewage disposal systems. Samples were taken in 1000 ml sterilized polypropylene bottles. All specimens were transported to the Environmental Microbiology Laboratory in the Islamic Azad University of Jahrom under cold conditions and stored at 4°C until the time of assay. A standard structured questionnaire was used to obtain information regarding the characteristics of the individual sewage samples (place, date, season and month).

### Concentration methods

The sewage samples were concentrated by using pellet and two-phase methods. The two-phase method was performed as described by Hovi et al. [17]. Briefly, the sewage sample was centrifuged for 10 min at 1500 g. Then 400 ml of the upper supernatant was concentrated with a mix of Dextran 20% (20 gr) (D5376, Sigma), Polyethylene Glycol6000, 30% (133.6 gr) (Merck) and NaCl 5 M, (16 ml) (Merck). After overnight incubation at 4°C in a separation funnel, a standard volume of 4 ml was harvested combining the bottom phase and the hazy interphase. The pellet method was carried out using a procedure suggested by Kargar et al. [18]. From the remainder of the sewage sample, 75 ml was transferred to 5 sterile centrifuge tubes and centrifuged at 5000 g for 10 min at 5°C. The tubes were kept at 4°C. Finally, in order to destroy bacteria and fungi, 1 ml of pure chloroform was added to 4 ml of the pellet and two-phase samples. This was followed by vigorous shaking and centrifugation at 200 and 2000 g for 20 and 10 min at 5°C, respectively. The upper aqueous phase was transferred to a sterile tube and kept at −20°C until use for the detection of group A rotaviruses.

### Rotavirus detection

All the concentrated specimens were tested for group A rotaviruses by enzyme immunoassay (EIA) (Rotavirus Ag ELISA, DRG, Germany), according to the manufacturer's instructions.

### Viral RNA extraction

Rotavirus dsRNA was extracted from concentrated samples using the RNX-Plus kit (CinnaGen, Tehran, Iran), according to the manufacturers protocol.

### Reverse transcription-polymerase chain reaction

Briefly, 5 μl of extracted RNA was added to a mix of DMSO, 5X RT buffer, dNTPs, primers Beg9, End9, and DW, denatured at 97°C for 5 min. Then reverse transcriptase and RNase inhibitor were added to make a final volume of 20 μl. The RT-PCR reaction was performed for 60 min at 42°C.

### Nested multiplex PCR for G genotyping

G-typing was performed according to the rotavirus detection and genotyping protocol provided by World Health Organization [19]. Briefly, 10 μ l of viral cDNA was added to a mix containing MgCl2 (50 mM), deoxynucleoside triphosphates (10 mM), 10x PCR buffer, Taq DNA polymerase (1U), the forward primer Beg9 (10 pmol) and the reverse primer End9 (10 pmol) to a final volume of 50 μl. The cycling parameters used were: 30 cycles at 94°C for 1 min, 42°C for 2 min, 72°C for 2 min, and a final extension at 72°C for 5 min. Five μl of first round VP7 amplicons were used as a template in the second round of PCR. The multiplex reaction mix also included each of the G-type-specific primers, aBT1 (G1), aCT2 (G2), aET3 (G3), aDT4 (G4), aAT8 (G8) aFT9 (G9), mG10 (G10) and G12. Finally, cycling was done with 20 cycles of the same cycling profile as the first reaction. The amplified product was analyzed by gel electrophoresis in 2% agarose gel containing ethidium bromide (10 μg/mL). The 100 bp DNA ladder (GeneRuler™, Fermentas life science) was used as a molecular weight standard. Primer sequences are shown in Table 1.

**Table 1:** **Primer sequences and positions used for genotyping of VP7 gene in rotavirus strains**[19]

**Type**	**Position**	**Sequence (5’→3’)**	**Primer**
-	nt 1-28	GGC TTT AAA AGA GAG AAT TTC CGT CTG G	Beg9
-	nt 1062-1036	GGT CAC ATC ATA CAA TTC TAA TCT AAG	End9
G1	nt 314-335	CAA GTA CTC AAA TCA ATG ATG G	aBT1
G2	nt 411-435	CAA TGA TAT TAA CAC ATT TTC TGT G	aCT2
G3	nt 689-709	CGT TTG AAG AAG TTG CAA CAG	aET3
G4	nt 480-498	CGT TTC TGG TGA GGA GTT G	aDT4
G8	nt 178-198	GTC ACA CCA TTT GTA AAT TCG	aAT8
G9	nt 757-776	CTA GAT GTA ACT ACA ACT AC	aFT9
G10	nt 666-687	ATG TCA GAC TAC ARA TAC TGG	G10 or mG10
G12	nt 548-567	CCG ATG GACGTAACGTTGTA	G12

### Statistical analysis

Data was statistically analyzed applying Chi-square, ANOVA or Fisher's exact test. The statistical software package SPSS version 18 (SPSS Inc., Chicago, IL, USA) was used for all statistical assessments. P values <0.05 were considered statistically significant.

## Results

**Figure 1:**
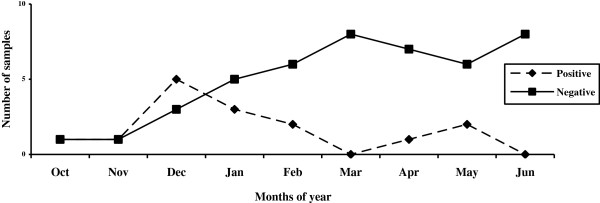
Rotavirus distribution from October 2010 to June 2011.

Rotaviruses were detected in 15 of 60 (25%) sewage samples that were analyzed for the presence of group A rotavirus antigen by EIA. Specifically, these included 73.33% (11 cases) of raw sewage influent samples and 26.67% (4 cases) of treated effluent samples. Overall, the analysis of the removal efficiency of the wastewater treatment plant revealed that the mean removal of rotaviruses in urban and hospital sewage disposal systems was 63.64% (50% in hospital effluent samples, and 80% in urban effluent samples) (Table 2). According to the seasonal distribution, it was revealed that rotaviruses were identified with a significantly higher frequency during the cold seasons of the year (*P*= 0.031). The highest prevalence of viruses was detected in autumn (46.67%), followed by winter (33.33%) and spring (20%), respectively. Rotaviruses were detected most frequently from December to January, representing 53.33% of all rotavirus-positive cases. The presence of rotaviruses remained low in March and June, in which no virus was detected. A significant relationship was also found between rotavirus detection and monthly distribution (*P*= 0.033) (Figure 1). Genotyping was performed on 15 rotavirus positive samples by using nested RT-PCR. The most common circulating genotype was the G1 type, identified in 11 out of 15 analyzed samples (73.33%), followed by G1G4 (20%) and non-typeable genotypes (6.67%), respectively. The genotypes G2, G3, G8, G9, G10 and G12 were not detected in this study. The most frequently detected genotype was G1 in the influent of the raw sewage samples (53.33%) and the effluent of the treated sewage samples (20.0%). No statistically significant differences between the genotype distribution and type of sewage samples was observed (*P*= 0.17) (Table 3). The most prevalent rotavirus genotype reported was G1 in spring (13.33%), autumn (40.0%) and winter (20.0%) seasons. Statistically significant differences were found for the distribution of the genotypes and seasons (*P*= 0.039).

**Table 2:** The frequency of collected samples and distribution of rotavirus in different sources of the wastewater treatment plants

	**Total samples (n=60)**		
	**Influent system**	**Effluent system**	**Efficiency rate†**
**Hospital**	16/6* (40.00%)	15/3* (20.00%)	50%
**Sewage**	15/5* (33.33%)	14/1* (6.67%)	80%
**Total**	31/11* (73.33%)	29/4* (26.67%)	63.64%

* The number of rotavirus positive samples.

**†** The removal efficiency= (The number of rotavirus positive in influent samples) - (The number of rotavirus positive in effluent samples)/The number of rotavirus positive in influent samples * 100.

**Table 3:** Distribution of rotavirus genotypes in different sources of the wastewater treatment plants

	**Hospital**		**Sewage system**		
**Genotypes**	**Influent system**	**Effluent system**	**Influent system**	**Effluent system**	**Total**
**G1**	5 (33.33%)	3 (20%)	3(20%)	-	11 (73.33%)
**G1G4**	1 (6.67%)	-	2(13.33%)	-	3 (20%)
N.T.A*	-	-	-	1 (6.67%)	1 (6.67%)
**Total**	6 (40%)	3 (20%)	5 (33.33%)	1 (6.67%)	15 (100%)

*Non typeable.

## Discussion

To our knowledge, this is the first study performed in Iran to detect group A rotaviruses in raw and treated sewage samples collected from urban and hospital wastewater treatment plants, located in the city of Shiraz, Iran. In the present study, we examined the prevalence, genotypes, seasonality and removal efficacy of rotaviruses in wastewater samples. There are high numbers of human pathogenic microorganisms present in sewage, including bacteria, viruses, and protozoan parasites. Therefore, raw sewage might be considered as an environmental contaminant and a major carrier of disease-causing agents, particularly enteric pathogens [4,12]. Enteric viruses, such as gastrointestinal tract pathogens, have the greatest significance in disease transmission by way of environmental water routes and are usually transmitted through the fecal-oral route by the consumption of contaminated water or food containing viruses and fecal waste from infected individuals [12,20,21].

Epidemiological studies have demonstrated human rotaviruses to be one the most predominant viral agents excreted into environmental waters in large numbers. In the present study, we found group A rotaviruses in 25% of the samples analyzed from raw and treated sewage. Our results are comparable to those of previous studies conducted around the world which have reported a prevalence of rotaviruses of 11 to 42% in wastewater samples [7,8,11,22].

This study demonstrated that there is a significant correlation between the seasonal profile and rotavirus-positive cases. Rotaviruses were detected most frequently in autumn and winter, with a peak virus load in December to January. Previous studies have also indicated a higher prevalence of rotaviruses during the cold months of the year in different types of environmental waters [11,23,24], corresponding to seasonal variations of rotaviral gastroenteritis in the population [14,15,25].

Most of the more than one hundred species of enteric viruses are difficult or impossible to detect using conventional cell-culture methods, such as human norovirus. Some viruses, such as rotavirus and the human hepatitis A virus, have a very poor detection efficiency [4,10,26]. For this reason, in recent years, molecular methods have rapidly found their way into environmental virology studies. Generally, different studies have considered PCR as a valuable technique for assessing the presence of viruses in environmental water samples. This is due to their high specificity and sensitivity in detecting even a few viral particles in environmental samples [10,26]. Even though PCR is a very sensitive detection technique, because of the low concentration of rotaviruses in environmental water, viral concentration methods could play a significant role in the improvement of intact virions versus naked genomes before the PCR can be attempted. Hovi et al. [17] demonstrated that the virus concentration in water samples can be increased up to 50±100-fold by using the two-phase method. Moreover, in the present study, the pellet and two-phase methods were used for enhancing the viral concentrations.

Analysis of rotavirus genotypes in this study showed that the most frequent circulating genotype in raw and treated sewage was the G1 genotype. The predominance of G1 strains in our study is in accordance with previously described clinical and environmental investigations worldwide [9,13,14,20,27,28]. The second most common genotype was the mixed genotype (G1G4), present in 20% of the evaluated samples. The high frequency of the mixed genotype with different rotavirus strains may reflect frequent contamination of water resources with rotavirus strains that facilitate the generation of novel rotavirus strains through a genetic re-assortment process. Therefore, the frequency of mixed infections with rotaviruses and its effect on the development of rotavirus vaccines should be thoroughly investigated. The non-typeable genotype was detected in only 6.67% (1/15) of the samples collected from the sewage effluent system and has not been detected in any of the samples collected from the plant’s influents. The non-typeable rotavirus strains for the G genotype could be related to the presence of novel strains or the failure of the genotyping due to the presence of other genes that was not investigated in this survey. For example rare genotypes such as G5, G6, G11 and a failure of RT-PCR techniques [19].

Najafi et al. [29] assessed the G genotypes of rotavirus circulating in clinical samples of children aged <5 years with acute gastroenteritis in Shiraz. In our study, a close genetic correlation was observed among detected genotypes in urban and hospital sewage systems and these clinical rotaviral isolates, demonstrating that rotavirus strains could have been disseminated into the environment and thus contaminated water resources. This contamination can facilitate the circulation of genotypes between the environment and the population as well as the generation of novel rotavirus strains through a re-assortment process. Thus, they could be considered as a reference for risk assessment.

Nowadays, sewage treatment systems play a significant role in the removal of human pathogens from wastewater. Therefore, an efficient treatment of sewage is crucial to the health of any community. In the current study, we assessed the efficiency of a wastewater treatment plant regarding the removal of rotaviruses. The data obtained in our study indicate that although the rotavirus detection rate was lower in samples collected from sewage plant effluents than in those collected from the influents, the virus contamination remained considerable (36.36%) and still represents a real public health hazard. This data is in agreement with the results of previous studies showing the presence of rotaviruses not only in raw, but also in treated wastewater [7,8,11]. Our removal efficiency results (63.64%) are comparable to previous reports in other parts of the world, suggesting that even properly working wastewater treatment systems remove only 20-80% of enteric viruses [8,11,30], confirming the high resistance of the virus to the sewage treatment process. The results obtained in this study demonstrate the greater efficiency of an urban wastewater treatment plant in eliminating the viral load (80%) as compared to a hospital sewage disposal system (50%). Because hospital wastewater is one of the most important sources of rotavirus contamination discharged into the environment, the effective management of hospital disposal systems in the elimination of pathogenic microorganisms should be seriously considered for the protection of the public health.

Typical bacterial indicators such as fecal coliforms, e.g. E.coli and Enterococci, are the indicators most often studied in order to examine the extent fecal contamination. In addition, they are utilized to evaluate the efficiency of human pathogen removal in water treatment processes. None of the bacterial indicators currently used are suitable for water quality monitoring programs because they are more sensitive to inactivation by water treatment processes than viral or protozoan agents. Their short survival rate was also taken into consideration and their ability to multiply in some environments as compared to other pathogens [26]. Different studies have revealed the resistance and stability of several types of viruses related to environmental stress and sewage treatment processes [3,22,24]. Because of this, during recent years, more attention has been paid to using viruses as an indicator of sewage contamination, the risk of waterborne viral diarrheal disease, and the necessity of routine surveillance of water sources for monitoring the viral contamination. Specifically, enteroviruses, noroviruses and rotaviruses have been proposed as indicators for monitoring the human fecal contamination of water and the efficacy of wastewater treatment procedures [31].

## Conclusion

The results obtained in the current study represent for the first time genotype information of rotaviruses circulating in the environment in Iran. The high frequency of group A rotaviruses in our study might be explained by the high survival rates of viruses in the wastewater treatment processes and also the relative inefficiency of wastewater treatment plants in eliminating these viruses. Therefore, regular viral monitoring should be considered as an additional analysis in the routine testing already performed in order to improve policies of wastewater management by the national water quality monitoring bodies. This viral testing should be added as part of microbial risk assessment and as a critical component in the evaluation of sewage quality. This preliminary study has highlighted the necessity of continuing strain characterization in other regions of Iran, in order to have a comprehensive picture of the geographic and temporal distribution of rotavirus strains circulating in the community. This may have important implications for rotavirus vaccine efficacy.

## Competing interests

The authors declare that they have no competing interests.

## Authors’ contributions

MK carried out the design of the study, coordination and helped to draft the manuscript. NJ participated in sampling, carried out the concentration and genotyping. AN carried out the immunoassay, Viral extraction and drafted the manuscript. YT participated in scientific consultation and performed the statistical analysis. All authors read and approved the final manuscript.
